# Peer Review of “Willingness to Pay for the COVID-19 Vaccine and Its Correlates in Bangladesh: Cross-Sectional Study”

**DOI:** 10.2196/79353

**Published:** 2025-08-15

**Authors:** Jatina Vij

**Affiliations:** 1 Post Graduate Institute of Medical Education & Research, Chandigarh, Madhya Marg, Sector 12, Chandigarh, India, 91 1722747585

**Keywords:** Bangladesh, willingness to pay, vaccines, COVID-19, infectious diseases, infection control, public health, public safety, cross-sectional study, financial

*This is a peer-review report for “Willingness to Pay for the COVID-19 Vaccine and Its Correlates in Bangladesh: Cross-Sectional Study.”*

## Round 1 Review

### General Comments

This paper [1] examines willingness to pay (WTP) for COVID-19 vaccines in Bangladesh using a cross-sectional survey. The integration of the health belief model and theory of planned behavior adds a theoretical foundation to the analysis. The study is well-structured, and the use of hierarchical logistic regression strengthens its analytical rigor.

However, several issues need to be addressed before acceptance. The sampling methodology raises concerns about representativeness, particularly due to the mix of online and face-to-face data collection. Additionally, some statistical interpretations require further clarification, and the discussion on policy implications could be expanded to provide actionable recommendations. Addressing these concerns will enhance the overall impact and credibility of the study.

### Specific Comments

#### Major Comments

1. The study employs both online and face-to-face data collection. However, the online survey may have overrepresented educated and tech-savvy individuals, while the face-to-face survey followed quota sampling.

2. Clarify the adjusted odds ratio interpretation. Some adjusted odds ratio values are close to 1, making practical significance questionable.

3. The impact of administrative divisions (eg, Sylhet having 4× higher WTP) should be further discussed. Are these differences due to economic, cultural, or policy variations?

4. While the study suggests subsidized vaccination programs, it would be helpful to compare findings with other low- and middle-income countries’ WTP trends.

#### Minor Comments

5. Ensure table captions clearly describe what is presented (eg, Table 2 should explicitly state that it presents logistic regression results).

6. Some sections contain grammatical errors and awkward phrasing (eg, “knowledge about the vaccine, vaccine process, conspiracy beliefs, behavioral practice, attitude toward a vaccine”; this list is repetitive and unclear).

## Abbreviations

WTP: willingness to pay
